# 

**Published:** 1851-12

**Authors:** 


### CONTENTS

### OF NO. VI.

---

### ORIGINAL COMMUNICATIONS.

### ESSAYS AND CASES.

ART. I.—The Sanitary Condition and Vital Statistics of Fayette County, Kentucky. By W. S. Chipley, M. D., Lexington, Ky., . . . . . . . 461
ART. II.—Statistical Tables. By W. L. Sutton, M. D., Georgetown, Ky., . . . . . . . . . . 490
ART. III.—A Lecture on Sanitary Reform. By Lewis Rogers, M. D., Louisville, Ky., . . . . . . . 497

### ORIGINAL INTELLIGENCE.

Lithotrity and Lithotomy, . . . . . . . . . . 535
American Medical Association—Committee on the Cure of Reducible Hernia, . . . . . . . . . . 539
Registration Law in Pennsylvania, . . . . . . . . 542
Index, . . . . . . . . . . . . . . 549

